# Phase II Study of Temozolomide and Thalidomide in Patients with Unresectable or Metastatic Leiomyosarcoma

**DOI:** 10.1155/2008/412503

**Published:** 2008-11-16

**Authors:** Michelle S. Boyar, Mary Hesdorffer, Mary Louise Keohan, Zhezhen Jin, Robert N. Taub

**Affiliations:** ^1^Department of Medicine, Memorial Sloan-Kettering Cancer Center, New York, NY 10065, USA; ^2^Mesothelioma Applied Research Foundation, Santa Barbara, CA 93190, USA; ^3^Department of Biostatistics, Mailman School of Public Health, Columbia University, New York, NY 10032, USA; ^4^Division of Medical Oncology, Department of Medicine, Columbia University Medical Center, Herbert Irving Pavilion, 161 Fort Washington Avenue, New York, NY 10032, USA

## Abstract

We assessed the efficacy of combined temozolomide and thalidomide in patients with unresectable or metastatic leiomyosarcoma in a phase II single-institution trial. Twenty-four patients were enrolled. Temozolomide (150 mg/m^2^/day for 7 days every other week) was administered with concomitant thalidomide (200 mg/day), and continued until unacceptable toxicity or disease progression. There were no complete responses and two (10%) partial responses. Five patients (24%) had stable disease for at least six months. Fourteen patients (67%) progressed after a median of two-month treatment. The median overall survival (twenty-two assessable patients) was 9.5 months [95% CI 7–28 months]. There were no treatment-related deaths or CTC grade 4 toxicities. Thirteen patients were dose-reduced or discontinued thalidomide due to toxicity. In conclusion, this combination of temozolomide and thalidomide provided disease stabilization in a subset of patients with advanced leiomyosarcoma. We hypothesize that temozolomide is the active agent in this regimen, and should be further studied.

## 1. INTRODUCTION

Soft tissue sarcomas are rare tumors, representing less than 1% of all new cancers diagnosed in the United States each year [1]. Complete surgical resection offers the best chance of cure for localized soft tissue sarcoma. Patients with unresectable or metastatic disease have a median survival of approximately 12 months. Chemotherapy is the main treatment option for these patients. Doxorubicin, ifosfamide, and dacarbazine (DTIC) each have single-agent activity with response rates approaching 20% [2–4]. When DTIC was added to doxorubicin, the response rate and overall survival increased at the cost of increased toxicity [5].

The use of immunohistochemistry and genetic markers to better define subsets of soft tissue sarcomas is changing the approach to clinical trial design in soft tissue sarcoma. There is increasing evidence that the different subtypes of soft tissue sarcoma have distinct biologic characteristics which define metastatic potential and response to therapy. The remarkable activity of imatinib in patients with gastrointestinal stromal tumors is proof of this principle [6]. Instead of large clinical trials which include a variety of histologic subtypes, newer trials are limiting enrollment to specific subtypes to assess response rates in a subset of patients. This has led to the identification of active regimens for certain subtypes of soft tissue sarcoma. However, new therapies are clearly needed for patients with advanced or metastatic leiomyosarcoma.

Over 50 subtypes of soft tissue tumors have been described in adults, and leiomyosarcomas are one of the most common malignant soft tissue sarcomas in adults. Leiomyosarcomas are derived from smooth muscle cells and can arise in any location. However, more than half are located in retroperitoneal or intraabdominal sites [7].

Temozolomide is a cytotoxic alkylating agent that was developed as an oral and less toxic alternative to DTIC. Both temozolomide and DTIC exert their antitumor effects through the formation of 5-3-methyl-1-triazenolimidazole-4 carboxamide (MTIC), the putative active chemical metabolite of DTIC [8, 9]. Temozolomide has activity against malignant gliomas and metastatic melanoma [10, 11]. Based on its similar mechanism of action to dacarbazine, temozolomide has been evaluated in soft tissue sarcoma using a variety of dosing schedules. In several studies which included a variety of STS subtypes, all of the clinical benefit was seen in patients with leiomyosarcoma [12–14].

Thalidomide is an agent shown to be useful in a variety of tumors. Its mechanisms of action in cancer may be multiple, including direct cytotoxic, antiangiogenic, and anti-inflammatory effects [15]. The combination of temozolomide and thalidomide has shown promising activity in metastatic melanoma [16] and metastatic neuroendocrine tumors [17], where it led to increased response rates when compared to temozolomide as a single agent. We conducted this single-institution phase II trial to assess the efficacy of a combination regimen of temozolomide and thalidomide in patients with unresectable or metastatic leiomyosarcoma.

## 2. PATIENTS AND METHODS

### 2.1. Study population

The study population consisted of adult patients (age ≥18) with histologically confirmed, locally advanced, unresectable or metastatic leiomyosarcoma. Prior treatment with up to three prior systemic chemotherapy regimens for advanced disease was permitted, as was previous dacarbazine treatment. Prior radiation therapy was allowed if completed at least 4 weeks prior to study drug administration. Patients were required to have at least one unidimensional measurable lesion documented on computed tomography (CT). Previously radiated lesions were excluded unless there was evidence of disease progression at that site prior to enrollment. Further inclusion criteria included: SWOG performance status of 0–2, life expectancy >2 months, adequate bone marrow function (absolute neutrophil count [ANC] ≥1500/*μ*L, platelets >70 000/*μ*L, and hemoglobin ≥10 g/dL), adequate hepatic function (bilirubin < upper limit of normal [ULN], AST or ALT <1.5 X ULN, alkaline phosphatase <2 X ULN or ≤5 X ULN with documented liver metastases), and adequate renal function (creatinine <1.5 X ULN, BUN <1.5 X ULN). Exclusion criteria included brain metastases, more than 3 prior chemotherapy regimens for treatment of leiomyosarcoma, insufficient recovery from toxicities of prior therapies, other serious medical or psychiatric illness, inability to take oral medications, prior malignancy other than curatively treated carcinoma in situ of the cervix or skin cancer, and pregnant or nursing women. Prior chemotherapy, radiation therapy or surgery must have been completed at least four weeks prior to enrollment. All patients and physicians participated in the Enhanced STEPS program (Enhanced System for Thalidomide Education and Prescribing Safety). All patients gave written informed consent before study entry. The study was approved by the local institutional review board committee and was conducted in accordance with the ethical principles stated in the Declaration of Helsinki and the guidelines on good clinical practice.

### 2.2. Study design

Patients received treatment with temozolomide at a dose of 150mg/m^2^/day orally on days 1 to 7 and days 15 to 21. Thalidomide was administered daily at a dose of 200 mg. No dose escalations were permitted. Cycles were repeated every 28 days. Temozolomide was held if ANC remained <1500/*μ*L or platelet count <100 000/*μ*L, and was not resumed until hematologic recovery. When treatment resumed, dose reductions for temozolomide were made based on nadir platelet count or ANC. For nonhematologic toxicities of grade 3 or higher as defined by the National Cancer Institute (NCI) Common Toxicity Criteria (CTC) grading system, dosage for the subsequent cycle of temozolomide was reduced to 125mg/m^2^ for grade 3 toxicity and to 100mg/m^2^ for grade 4 toxicity. No dose adjustments were required if the toxicity was judged to be non-drug-related. Treatment was discontinued if the patient was unable to resume therapy within three weeks or if they experienced unacceptable toxicity levels. Dose modifications for thalidomide were made based on thalidomide-related toxicity. When thalidomide-related toxicity was noted, the dose of thalidomide was reduced to 100 mg daily; patients who experienced toxicity at a dose of 100 mg were taken off thalidomide.

Radiologic assessments of all sites of measurable disease with CT scan were performed on enrollment and every 8 weeks after starting treatment. Therapy was continued until evidence of disease progression on CT scan, unacceptable drug toxicity, delay of both drugs for >21 days, or patient-initiated withdrawal for any reason. Standard World Health Organization (WHO) response criteria were used. A complete response (CR) required complete disappearance of all clinically detectable malignant disease for at least four weeks. A partial response (PR) required ≥50% decrease in the sum of the products of the largest perpendicular diameters of a measurable lesion. Not all lesions had to show regression to qualify for partial response, but no lesion should have increased by ≥25%, and no new lesions should have appeared. Stable disease (SD) was defined as <50% decrease or <25% increase in the sum of the products of the largest perpendicular diameters of all measurable lesions. Progression of disease (PD) was defined as a ≥25% increase in the sum of products of measurable lesions, clear worsening of any evaluable disease, or appearance of any new lesions.

### 2.3. Statistical analysis

This was an open-label, phase II study conducted at Columbia University Medical Center. The primary end point in this trial is response (complete and partial response) rate. A response probability of 25% would be of interest, while further testing would not be pursued if the response probability was 5% or lower. Initially, 15 patients would be entered. If at least one response was observed, an additional 10 patients will be entered into the trial. Four or more responses out of a total of 25 patients would be considered as evidence warranting further study of this regimen, provided that other factors, such as toxicity and survival were favorable. This design has a significance level (probability of falsely declaring an agent with a 5% response probability to warrant further study) of 5%, and a power (probability of correctly declaring an agent with a 25% response probability to warrant further study) of 90%.

Time to progression, overall survival, safety, and toxicity were assessed as secondary outcomes. Time to progression (date of initiation of treatment to date of progression or death) and overall survival (time from treatment initiation to the date of death) were assessed with Kaplan-Meier estimator. Toxicity and complications of treatment were assessed based on patient reports of adverse events, physical examination, and laboratory measurements.

## 3. RESULTS AND DISCUSSION

### 3.1. Patient characteristics

A total of 25 patients were entered on the study from February 2002 to November 2004. One patient was found to be ineligible and withdrawn from the study before receiving therapy; that patient was excluded from the analysis. Demographics and baseline disease characteristics for the 24 treated patients are presented in Table 1. Patients had a median age of 60 years (range from 27 to 75 years); 22 (92%) were females and 2 (8%) males. Median SWOG performance status was 1. All patients had biopsy-proven leiomyosarcoma. The primary site was uterus in 11 (46%) of patients. Other primary sites included retroperitoneum (*n* = 3, 13%), small or large intestine (*n* = 3, 13%), ovarian (*n* = 1, 4%), and unknown (*n* = 3, 13%). All patients had evidence of metastatic disease. Fifteen patients (63%) had lung metastases and fourteen patients (58%) had liver metastases. Eight patients (33%) had metastases to bone or soft tissue. Twenty patients (83%) had received prior chemotherapy, including treatment with doxorubicin (*n* = 16, 67%) and/or dacarbazine (*n* = 4, 17%) and others. The median number of prior regimens was 2. Fourteen patients (58%) had prior surgery, and three (13%) had prior radiation therapy.

### 3.2. Duration of treatment

Of the 25 patients consented for the trial, 24 patients received treatment for a median of two months (range from 0.5 to 25 months) (Table 2). Five patients received treatment for 6 months or more. Three patients received treatment for less than 1 cycle due to rapidly progressive disease (*n* = 2), and withdrawal of consent (*n* = 1). Two patients required dose reduction of temozolomide due to neutropenia and thrombocytopenia. Thirteen patients required dose reduction of thalidomide (*n* = 8) or discontinued thalidomide (*n* = 5) due to neuropathy or fatigue.

### 3.3. Toxicity

Twenty one patients were assessable for toxicity, which is summarized in Table 3. Grade 2 or 3 neutropenia developed in three patients (12%), and grade 2 or 3 thrombocytopenia developed in 4 patients (17%). Dose reduction of temozolomide was required in 3 patients. Grade 2 or 3 anemia was seen in 3 patients. There were no grade 4 hematologic toxicities.

Fatigue was the most common nonhematologic toxicity with grade 2 or 3 fatigue occurring in 13 (54%) patients, and attributed primarily to thalidomide toxicity. Grade 2 or 3 nausea and emesis occurred in 8 (33%) and 10 (41%) of patients. Neurologic toxicity occurred in 8 patients and was primarily neuropathy, however, one patient had grade 3 vision loss, and two patients had grade 3 ataxia. Other toxicities were relatively mild and consisted of grade 2 anorexia in five patients (21%), grade 2 constipation in 10 patients (42%), and grade 2 edema in three patients (13%). There were no infectious complications due to treatment.

### 3.4. Efficacy

**Figure 1:**
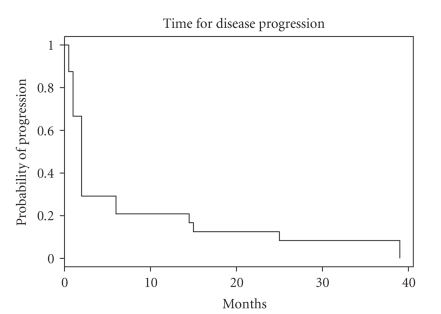
Time for disease progression.

**Figure 2:**
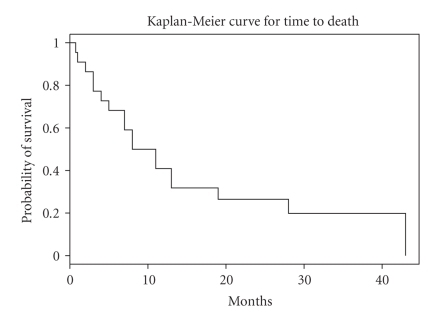
Overall survival.

Twenty one patients completed at least one cycle and were assessable for treatment response. There were no complete responses. Two patients experienced durable partial responses. The overall radiologic response rate was 10%. Five (24%) patients experienced stable disease, and 14 (67%) had disease progression. The two patients with radiographic responses had durable responses lasting 24 and 25 months before disease progression. For the five patients who had disease stabilization, the median duration of stable disease was 15 months (range from 6 to 24 months). The median follow-up time for the patient cohort is 41 months (range from 18 to 51 months). Twenty three patients developed progressive disease while receiving therapy, and the median progression-free survival was 2 months with 95% CI (2 months–6 months) as shown in Figure 1. Twenty two patients are assessable for survival and the median overall survival for the cohort is 9.5 months with 95% CI (7 months–28 months). The one-year survival rate was 40.9% with 95% CI (24.8%, 67.6%), and the 2-year survival rate was 26.5% with 95% CI (13.1%, 53.9%) (Figure 2).

The results of this study suggest that the combination of temozolomide given on an alternating weekly schedule with daily thalidomide has minimal clinical activity in patients with locally advanced or metastatic leiomyosarcoma. Two (10%) out of 21 patients evaluable for response had a partial response by WHO response criteria. Five patients (24%) had disease stabilization for at least 6 months while receiving treatment. The overall response rate in this study is lower than what has been reported with DTIC alone [4]. Despite the low response rate, both patients on this trial with radiographic responses had durable responses lasting 24 and 25 months before disease progression. The median survival of 9.5 months is typical for this advanced stage, heavily pretreated patient population.

Temozolomide has been evaluated in soft tissue sarcoma using several different dosing schedules. In our institution, a phase II trial enrolled 26 patients with unresectable or metastatic soft tissue sarcoma who were treated with temozolomide administered twice daily on a 12-hour schedule for 5 days as an oral bolus dose of 200 mg/m^2^ followed by 9 doses of 90 mg/m^2^ every four weeks. There were 2 partial responses, 2 mixed responses, and 3 patients with stable disease lasting >6 months, for an overall objective response rate of 8%. All of the patients with clinical benefits had leiomyosarcoma, none of the other soft tissue sarcoma histologic subtypes had any benefit [12]. Another phase II study was conducted in 60 soft tissue sarcoma patients (19 with GIST and 41 with other STS histologies). There were no responses seen in the patients with GIST, and 22% had stable disease. Of the evaluable patients with other soft tissue sarcomas, there was 1 CR and 1 PR for a total response rate of 5%, another 33% had stable disease. The median time to progression and median overall survival time in patients with other STS was 3.3 months and 11 months [13]. A phase II study by the EORTC treated 31 patients with advanced STS with temozolomide dosed at 750 mg/m^2^ over 5 days during cycle 1 and then 1000 mg/m^2^ over 5 days at cycle 2. There was only 1 partial response for an overall response rate of 3.33%. The median TTP was 2 months and the median OS was 6.75 months [14].

The Spanish Group for Research on Sarcomas conducted a phase II trial of temozolomide given as daily for 6 weeks at a dose of 75 mg/m^2^/day–100 mg/m^2^/day. They enrolled 49 patients with pretreated STS and 18 patients with GIST. Among the patients in the STS arm, there were 7 PR for an overall response rate of 15.5%. There were 11 patients with gynecologic leiomyosarcoma enrolled, 5 of which showed response. The median response duration was 12.5 months (range from 3.9 to 58 months). In 4 patients the response lasted over one year. The median TTP was 2.2 months and median OS was 8.1 months. The drug was well tolerated at this dose and grade 3-4 hematologic toxicities were seen in 10–15% of patients. This suggested that the extended daily dosing schedule had activity in patients with gynecologic leiomyosarcoma [18]. Memorial Sloan-Kettering published their experience with temozolomide in patients with pretreated leiomyosarcoma from 2001 to 2004. Twelve patients were treated with continuous daily dose temozolomide, there was one PR which lasted 4 cycles and 4 patients had stabilization of disease from 2 to 5 cycles. Seven patients were treated with bolus dose temozolomide. One patient had a near CR which lasted 13 cycles and four patients had disease stabilization lasting from 3 to 16 cycles [19]. The collective evidence from these phase II trials suggested that leiomyosarcomas, particularly uterine leiomyosarcomas may be more responsive to treatment with temozolomide than other STS histologic subtypes.

In our study, enrollment was limited to patients with leiomyosarcoma, but patients with leiomyosarcoma originating from any site were eligible. Although there were only two partial radiographic responses among the evaluable patients (10%), the duration of response for both patients was prolonged, lasting 24 and 25 months, respectively. Other groups have also reported patients with durable responses lasting over 12 months [18]. The significance of this finding in a disease where the median progression-free survival ranges from two to three months suggests there is a cohort of leiomyosarcoma patients who may derive significant benefit from treatment with temozolomide.

The relative contribution of thalidomide to the antitumor efficacy in this study is difficult to determine. Although the reported toxicities were generally mild, grade 2 thalidomide-related toxicities such as fatigue, constipation, and neurologic toxicity were seen in up to 50% of patients. Dose reduction or discontinuation of thalidomide was required in 13 (54%) of patients. Patients who continued treatment with temozolomide had continued benefit even after stopping the thalidomide. We concluded that thalidomide was poorly tolerated, and it is unlikely to add additional antitumor efficacy in this patient population. Our results and those of other groups suggest that temozolomide is the active agent in this regimen.

## 4. CONCLUSIONS

In conclusion, temozolomide offers another option to consider in the treatment of patients with advanced or metastatic leiomyosarcoma. Its benefits include the convenience of oral administration and an improved side-effect profile compared to traditional chemotherapy regimens for advanced soft tissue sarcoma. Although it does not produce high response rates, there are a number of patients who have disease stabilization from months to years even without radiographic responses. There are very few chemotherapy regimens that offer the possibility of benefit in this patient population, and our data support the consideration of temozolomide in the treatment of progressive disease. Although several dosing schedules have been tested in trials (continuous daily dosing, bolus dosing and biweekly dosing), there is not sufficient evidence to support adopting an alternative dosing schedule, therefore, we recommend using the 5-day bolus dosing regimen for temozolomide.

Future investigation should focus on determining the biologic and molecular characteristics of leiomyosarcomas that can predict response to temozolomide. Methylation of the DNA repair protein 06-methylguanine-DNA-methyltransferase (MGMT) has been identified as a predictor of response to temozolomide treatment in patients with glioblastoma multiforme [20]. Epigenetic silencing of MGMT and/or other key regulatory genes in tumor cells may play a role in temozolomide resistance, and in the pathogenesis of soft tissue sarcomas. Because of the potential therapeutic benefits to those patients who may respond to temozolomide, investigation of mechanisms of response and resistance to this drug warrants further investigation in leiomyosarcomas.

## Figures and Tables

**Table 1:** Baseline patient characteristics.

Characteristics	Patients (*N* = 24)	Percent
	No.	(%)
Age, years		
* *Median	60	
* *Range	27–75	
Sex		
* *Male	2	8
* *Female	22	92
SWOG performance status		
* *0	1	4
* *1	18	75
* *2	5	20
Histology		
Leiomyosarcoma	24	100
Primary site		
* *Uterus	11	46
* *Retroperitoneum	3	13
* *Other	10	42
Sites of metastases		
* *Lung	15	63
* *Liver	14	58
* *Other	8	33
Prior treatment		
* *Surgery	14	58
* *Radiotherapy	3	13
* *Chemotherapy	20	83
* * Doxorubicin	15	67
* * Dacarbazine	4	17
No. of prior chemo regimens		
* *0	4	17
* *1	6	25
* *2	9	38
* *3	5	21

**Table 2:** Response, location of primary disease, prior therapy, and duration of therapy (days).

Enrolled patient ID No.	Response	Primary site of disease	Prior chemotherapy regimens	Duration of therapy (days)
1	PD	Colon and duodenum	Dox	34
2	PD	Uterus	Dox/taxotere; Ifos/DTIC/etoposide; gleevec	16
3	PD	Uterus	Dox/ifex; gem/taxotere	37
4	PD	Stomach	Gleevec; ifex/dox	8
5	PD	Retroperitoneal	Doxil; ifos; taxotere/gem; digitoxin	25
6	**SD**	Unknown	Dox/ifex	*114/398
7	PD	Iliopsoas	Dox; gem/taxotere	42
8	**PR**	Retroperitoneal	None	*382/389
9	PD	Ovarian	Doxo/ifex; gem/taxotere	45
10	PD	Uterus	Doxil, gem	62
11	PD	Uterus	Gem/taxotere	56
12	PD	Pelvic mass	Gem/taxotere	*36/62
13	**PR**	Uterus	Dox/ifos; gem/taxotere	670
14	PD	Unknown	DTIC/doxo; gem/taxotere; digitoxin; ifos	50
15	**SD**	Uterus	Dox/ifox/taxotere	186
16	PD	Uterus	Gem/taxotere	52
17	**SD**	Uterus	Dox; gem; taxotere	143
18	**SD**	Uterus	None	*157/207
19	PD	Colon	None	37
20	PD	Uterus	Ifos; gem	*30/53
21	PD	Kidney	None	78
22	PD	Small bowel	Dox/avastin; gem; DTIC	53
23	**SD**	Retroperitoneal	MAID; vin/doxo/cytoxan	383
24	PD	Uterus	Gem/taxotere	36

*For the 5 patients who discontinued thalidomide early, the fraction represents duration of therapy in days for Temodar + Thalidomide over total days of therapy with Temodar.

**Table 3:** Most common or serious hematologic and nonhematologic toxicities.

Toxicity	Grade 2		Grade 3		Grade 4	
	No.	%	No.	%	No.	%
Hematologic						
* *Neutropenia	2	8	1	4		
* *Thrombocytopenia	3	13	1	4		
* *Anemia	2	8	1	4		
Non-hematologic						
* *Nausea	8	33				
* *Emesis	8	33	2	8		
* *Fatigue	12	50	1	4		
* *Neurologic toxicity	5	21	3	12		
* *Constipation	10	42				
* *Anorexia	5	21				
* *Edema	3	13				
